# Gangrene due to axial torsion of a Giant Meckel's Diverticulum containing multiple stones in the lumen: a case report

**DOI:** 10.1186/1757-1626-2-7141

**Published:** 2009-05-18

**Authors:** Quentin M Nunes, Alex Hotouras, Sandeep Tiwari, Anuradha Sheth

**Affiliations:** 1 Department of Surgery, Lincoln County HospitalLincolnUK; 2 Department of Histopathology, Lincoln County HospitalLincolnUK

## Abstract

Meckel's diverticulum is the most common congenital anomaly of the small intestine. Common complications related to a Meckel's diverticulum include haemorrhage, intestinal obstruction and inflammation. Gangrene due to axial torsion and enteroliths of a Meckel's diverticulum are the rarest complications that have been reported in the literature. We report a case of gangrene due to axial torsion of giant Meckel's diverticulum with multiple stones in its lumen.

## Introduction

Meckel's diverticulum is the most common congenital anomaly of the small intestine, with a prevalence of approximately 2%. Symptoms resulting from a Meckel's diverticulum occur because of complications such as haemorrhage, intestinal obstruction and inflammation [1]. Gangrene due to axial torsion and enteroliths are the rarest complications of a Meckel's diverticulum and have not been reported together in the same patient.

## Case presentation

**Figure 1.**
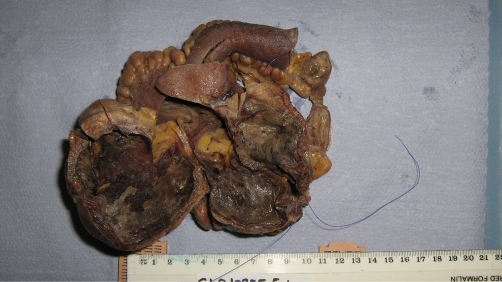
Resected specimen showing Giant Meckel's diverticulum.

**Figure 2.**
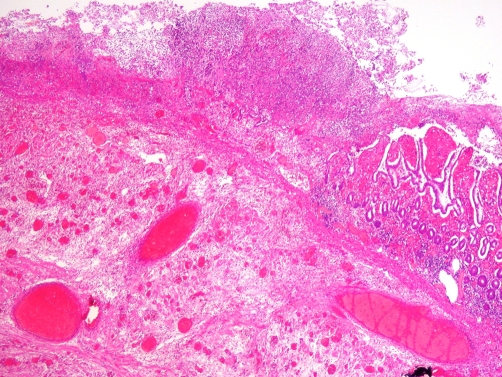
Histological image of Meckel's diverticulum.

A 47 year old Caucasian man presented to our hospital as an acute admission with two days history of colicky central abdominal pain associated with diarrhoea and a temperature of 37.8 °C. He was otherwise fit and healthy and had no history of any abdominal surgery in the past. On clinical examination, there was tenderness in the right lower abdomen with local peritonism. The white cell count was elevated at 12.6 × 10^9^ /L with a neutrophilia (>90%) and the C-reactive protein titre was 114 mg/L. All other laboratory markers were within normal limits. An ultrasound scan of the abdomen revealed a 6 cm × 5 cm × 4.6 cm fluid filled area containing echogenic components in the right iliac fossa with a trace of free fluid surrounding it. A diagnosis of acute appendicitis was made and an emergency appendicectomy was planned. At operation, a copious amount of turbid fluid in peritoneal cavity was noted while the appendix and caecum were normal. The operating surgeon proceeded to a lower midline laparotomy, which revealed an inflamed, oedematous, antimesenteric diverticulum with a twist around its base and a gangrenous tip. It measured approximately 140 cm × 30 cm (Figure 1) and was located approximately 80 cm from the ileocaecal valve. Resection of the involved small bowel segment with a primary side to side stapled anastomosis was performed. Histology confirmed a torted Meckel's diverticulum lined by heterotopic gastric mucosa with multiple stones in the lumen (Figure 2). The patient recovered well without any complications.

## Discussion

Meckel's diverticulum is a true diverticulum located in the distal ileum, usually within 60-100 cm of the ileocaecal valve. It is typically 3-5 cm long, runs along the antimesenteric border of the small bowel and has its own blood supply. It is a remnant of the vitellointestinal duct that may occasionally contain heterotopic gastric mucosa [2]. Although Meckel's diverticulum is the most common congenital anomaly of the small intestine [2], gangrene due to axial torsion and the formation of calculi within such diverticuli are considered the rarest complications [3,4]. Although the two above mentioned complications have been reported separately in the literature in few cases, they have never been reported together in the same case. In our case, gangrene due to axial rotation of Meckel's diverticulum and multiple stones in the lumen were identified in the same patient. The majority of these diverticuli appear to be associated with two characteristics: 1) a wide neck and 2) the presence of smooth muscle which is capable of peristalsis. The presence of these two factors renders the formation of enteroliths very difficult because they do not favour the stagnation of intestinal contents within the diverticulum [3]. However, stasis may still occur if a flap of diverticular mucosa functions like a one-way valve and prevents drainage of contents [3]. In addition, the presence of inflammation and oedema around the neck of the diverticulum may lead to narrowing of the opening and decrease drainage precipitating the formation of calculi [5].

Although patients with Meckel's diverticuli are usually asymptomatic, they carry a 4-6% lifetime risk of developing a complication such as haemorrhage or inflammation [2,6]. Epidemiological studies have shown that there is no relation between the incidence of complications and the age of the patient. However, males are more prone to developing a complication that females and, hence, more likely to be diagnosed with Meckel's related complications [6,7].

A high index of suspicion is required in order to make the diagnosis of Meckel's diverticulum promptly since any delay may lead to significant morbidity and mortality. Radiological investigations such as plain abdominal radiograph, sonography and computed tomography may be used to assist the diagnosis of symptomatic Meckel's diverticulum but, in practice, the rarity of the enteroliths means that they are often unreported [5]. Scintigraphic (^99^Tc^m^-pertechnetate) localisation of gastric mucosa may be used to facilitate the diagnosis of Meckel's diverticulum [8]. Since appendicitis is a far more common differential diagnosis, inspection of the last meter of small bowel during appendicectomy, particularly if the appendix does not appear to be inflamed, should be considered mandatory.
